# UK Foundation Programme training needs a national minimum standard for advanced life support

**DOI:** 10.1016/j.clinme.2026.100605

**Published:** 2026-06-08

**Authors:** Adam Baker, Michael Okorie

**Affiliations:** a Royal Sussex County Hospital, University Hospitals Sussex NHS Foundation Trust, Brighton, United Kingdom; b Brighton and Sussex Medical School (BSMS), Brighton, United Kingdom

**Keywords:** Advanced life support, Foundation training, Resuscitation, Medical education, Patient safety, Prescribing safety assessment

## Abstract

Since 2021, advanced life support (ALS) has no longer been a mandatory requirement of the UK Foundation Programme. Although the shift towards capability rather than certification was educationally defensible, in practice it has created marked national variation in how resuscitation preparedness is developed, evidenced and signed off. Foundation doctors now enter emergencies with unequal access to funded ALS training, simulation and supervised assessment, while supervisors are left to judge equivalence without a consistent national threshold. This matters not because patient-level harm has been neatly proven, but because high-stakes clinical capability should not depend on local opportunity or variable interpretation. A capability-based curriculum and a national minimum standard are not mutually exclusive. The Foundation Programme should therefore restore a national ALS requirement, or a formally recognised equivalent standard, alongside funded access, blended delivery and transparent national evaluation of uptake, exemptions and outcomes.

In 2021, advanced life support (ALS) ceased to be a mandatory requirement of the UK Foundation Programme. The curriculum shifted towards capabilities rather than prescriptive certifications, and the UK Foundation Programme Office (UKFPO) made clear that foundation doctors were still expected to obtain and demonstrate acute care skills, with ALS remaining ʻa very good way’ to learn and evidence them.^1^ On paper, that is a defensible educational principle. In practice, however, it created a problem. Resuscitation capability is still expected nationally, but access to the most widely recognised route for acquiring and demonstrating it is now determined locally. For a skill set that is high stakes, time critical and delivered in the most pressured moments of hospital care, that is not a minor administrative change. It is the creation of avoidable variation.

That variation is now the central issue. The post-2021 system is not one of ʻno ALS’, but one in which preparedness depends heavily on local opportunity. In the best settings, foundation doctors have access to funded courses, regular simulation and supervisors who directly observe and debrief emergencies. In the worst, study leave is difficult, simulation is sporadic and resuscitation capability is inferred from portfolio entries rather than standardised training or assessment. That matters because Annual Review of Competence Progression (ARCP) still requires evidence of relevant life support capabilities, and the curriculum still expects foundation doctors to demonstrate proficiency in the care of acutely unwell patients.^2^, ^3^ What has replaced a universal minimum standard is not a better national alternative, but a patchwork of local attempts for equivalencies.

The problem is not only access to training, but also how capability is being signed off. If an ALS certificate is no longer required, progression depends on supervisors being satisfied that equivalent resuscitation capability has been demonstrated through workplace evidence, simulation and portfolio review. That may be reasonable for many curriculum outcomes. It is less convincing for a rare, high-consequence skill such as advanced resuscitation. Educational supervisors are still asked to confirm broad progression across the curriculum, yet many will never have directly observed a foundation doctor participating in a peri-arrest or cardiac arrest call at the level ALS is designed to teach. Some can make that judgement from first-hand observation; many cannot. That is not a criticism of supervisors. It is a criticism of a system that has delegated assurance to variable local exposure without defining a dependable common standard against which that exposure can be judged.

The strongest counterargument is that mandatory certification is a blunt educational tool. A certificate does not guarantee real-world performance, and formal courses can crowd out better local learning such as repeated multidisciplinary simulation, contextual rehearsal and debrief. That argument deserves to be taken seriously. But it does not justify the current position. The real choice is not between national certification and meaningful workplace education. The better model is both: a defensible national threshold, combined with local reinforcement. Foundation training should not use ALS as a substitute for supervised clinical learning; it should use it as the floor beneath which preparedness should not fall.

The safety case remains important, even if it is difficult to prove neatly through patient-level outcomes. The European Resuscitation Council continues to recommend accredited advanced life support training for healthcare professionals and links resuscitation education with improved processes and outcomes.^4^ The best available systematic review found that participation in advanced life support courses was associated with improved return of spontaneous circulation and a favourable signal for survival.^5^ The obvious objection is that arrest outcomes are too heterogeneous to attribute to training alone. That is true, but it is not reassuring. When outcomes are shaped by recognition of deterioration, staffing, defibrillator access, Do Not Attempt Cardiopulmonary Resuscitation (DNACPR) practice, intensive care unit (ICU) admission and post-arrest care, the absence of a neat signal cannot be taken as evidence that variable training is harmless.

National in-hospital cardiac arrest data illustrate that problem. Survival to discharge rate after treated in-hospital cardiac arrest in the UK is reported at 25.8%, compared with 23.6% in earlier UK summaries.^6^, ^7^ On the surface, that seems reassuring. But those figures are system indicators, not training indicators. The relevant question is not whether national survival has collapsed since ALS stopped being mandatory for foundation doctors. It has not. The question is whether the policy has introduced avoidable variation in preparedness, escalation and team contribution among some of the most junior medical responders in hospital. At present, we cannot answer that confidently. For a patient-safety issue, that uncertainty should be uncomfortable rather than acceptable.

The inconsistency becomes harder to defend when compared with prescribing. The Foundation Programme already accepts that some high-risk activities justify national standardisation. The ARCP checklist explicitly requires successful completion of the Prescribing Safety Assessment (PSA) for completion of Foundation Year 1 (FY1).^2^ That reflects a simple principle: where an activity is safety critical and error prone, a national threshold is justified, and often the associated assessment drives learning. Resuscitation is less frequent than prescribing, but arguably even less forgiving. Both require rapid decision making, application of algorithms under pressure and the ability to act safely in situations where error can cause immediate harm. Yet one is protected by a national pass requirement, and the other is left to variable local provision and portfolio evidence. If the programme accepts the principle of national standardisation for prescribing, it is difficult to explain why resuscitation preparedness should be treated differently.

Confidence also matters, and not as a soft or secondary outcome. ALS is not simply basic life support with an advanced certificate attached. It encompasses rhythm recognition, safe defibrillation, airway management, care of the deteriorating patient, leadership, teamwork and post-resuscitation care.^4^ These are not peripheral skills. They are central to the management of deteriorating hospital patients. Foundation doctors often form part of the medical emergency and cardiac arrest teams, and they may be expected to perform roles that go well beyond compressions and calling for help. Evidence on confidence is limited and not UK-specific, but a recent study of junior doctors found a familiar pattern: confidence was high for basic cardiopulmonary resuscitation (CPR) tasks and lower for advanced interventions such as defibrillation and administration of resuscitation drugs.^8^ Those are precisely the parts of resuscitation least safely inferred from generic exposure alone.

ALS remains a curriculum requirement within internal medicine training (IMT). That does not justify deferring it from foundation training. Foundation doctors may not always lead arrests, but they form a substantial part of the resuscitation team. The same is true of emergency department (ED) nurses, many of whom hold ALS to gain the situational awareness, structured communication and the A to E approach the course teaches. These skills are not medicine specific: a surgical foundation trainee managing a deteriorating postoperative patient uses the same A to E approach as one on a medical take. There is also a fairness point. ALS now features on shortlisting criteria for many post-foundation trust-grade and clinical fellow posts; in the current market, that can be the difference between interview and unemployment. A system where some foundation doctors finish foundation training with ALS and others do not because of where they trained is hard to defend.

The practical case for a national minimum standard is stronger than it first appears. A mandatory model does not require every doctor to attend an inflexible two-day course in identical circumstances. The standard could be delivered through ALS, blended electronic ALS (e-ALS) or a formally recognised equivalent pathway, provided the endpoint is nationally defined and externally defensible. NHS England already routes e-ALS access for foundation doctors through employing trusts and allows reimbursement within prescribed limits.^9^ The infrastructure for a more standardised model therefore already partly exists. The main challenge is not conceptual feasibility but prioritisation. In health-economic terms, a nationally specified course is also easier to defend than the hidden cost of repeated local workarounds: duplicated ad hoc teaching, inconsistent supervisor assurance, variable study leave decisions and the remediation that follows when uncertainty is discovered late.

What should change is straightforward. By the end of Foundation Year 2 (FY2), every foundation doctor should either hold ALS or have met a nationally recognised equivalent standard with the same practical and cognitive expectations. Access should be funded, protected and planned early enough to avoid last-minute inequity. Local simulation should reinforce formal training, not replace it. And if policymakers remain unconvinced, then the least they should do is evaluate the current system properly. Foundation schools or NHS England should publish data on uptake, completion, delay, exemption and any association with ARCP capability sign-off. Five years after ALS was removed from the mandatory foundation curriculum, the most uncomfortable finding is not that national outcomes have clearly worsened. It is that we still cannot confidently describe the consequences. We have created a system in which access, exposure and credibility of sign-off vary, while the capability itself remains essential. For something as high stakes as resuscitation, that uncertainty is not a reason to wait. It is the reason to act. Foundation training needs a national minimum standard for advanced life support.

## CRediT authorship contribution statement

**Michael Okorie:** Writing – review & editing, Supervision, Conceptualization. **Adam Baker:** Writing – review & editing, Writing – original draft, Investigation, Conceptualization.

## Funding

This research received no specific grant from any funding agency in the public, commercial or not-for-profit sectors. As an opinion article based on synthesis of published literature and professional experience, no external funding was sought or required for its preparation.

## Declaration of Competing Interest

The authors declare the following financial interests/personal relationships which may be considered as potential competing interests: Adam Baker reports a relationship with Resuscitation Council UK that includes: non-financial support. Adam Baker is a qualified advanced life support (ALS) instructor with the Resuscitation Council UK. This role is disclosed because it relates directly to the subject matter of this article. Adam Baker confirms that this relationship did not influence the conclusions drawn. Michael Okorie declares that he is a clinical and educational supervisor, including a clinical supervisor of Adam Baker. If there are other authors, they declare that they have no known competing financial interests or personal relationships that could have appeared to influence the work reported in this paper.
